# OP06 Utilizing Health Technology Assessment Outputs To Develop Health Technology Management Protocols In The Irish Setting

**DOI:** 10.1017/S0266462324000709

**Published:** 2025-01-07

**Authors:** Claire Gorry, Maria Daly, Rosealeen Barrett, Karen Finnigan, Amelia Smith, Stephen Doran, Bernard Duggan, Sarah Clarke, Michael Barry

## Abstract

**Introduction:**

Increasingly in Ireland, there are specific criteria attached to reimbursement approval for new medicines. Health technology assessment (HTA) identifies where uncertainty is greatest in relation to clinical and cost-effectiveness evidence and budget impact estimates; our health technology management (HTM) approach uses these outputs from HTA to design protocols to manage these uncertainties in the post-reimbursement phase.

**Methods:**

A bespoke managed access protocol (MAP) is developed for each medicine reimbursed under this approach, informed by uncertainties highlighted in the HTA, directions from the decision-maker, and relevant particulars arising from commercial negotiations. Individual patient reimbursement applications are submitted via an online application system linked directly to the national pharmacy claims system. Pharmacists review the applications and approve reimbursement support where the patient meets the reimbursement criteria. The process is adaptive, allowing expansion of the criteria to include previously excluded patient cohorts, and the addition of new indications. It can also work across differing reimbursement arrangements (hospital/primary care).

**Results:**

The MAP for liraglutide for weight management confines reimbursement to patients with a body mass index greater than or equal to 35 kg/m², prediabetes, and high risk for cardiovascular disease. Phase I reimbursement support lasts for six months; patients not attaining greater than or equal to five percent weight loss are deemed non-responders as per the HTA, and reimbursement support is discontinued. The MAP for dupilumab confined reimbursement support to adults with refractory moderate-to-severe atopic dermatitis, where cost-effectiveness was plausible in the HTA. The MAP for calcitonin-gene-related-peptides monoclonal antibodies confines reimbursement support to patients with chronic migraine, refractory to at least three prophylactic treatments, where cost-effectiveness was plausible in the HTA.

**Conclusions:**

Across these MAPs, over 3,000 patients accessed novel treatments for chronic illnesses in September 2023. HTM provides an effective mechanism to facilitate access to high-cost medicines for targeted patient groups, while providing increased oversight and budgetary certainty. Key to acceptance is utilization of HTA outputs to implement evidence-based HTM measures targeting specific uncertainties as highlighted in the HTA report.

